# Influence of a 7 T magnetic field on growth, biomineralization, and denitrification metabolism in *Magnetospirillum gryphiswaldense* MSR-1

**DOI:** 10.1128/aem.01069-25

**Published:** 2025-09-17

**Authors:** Jing Zhang, Juan Wan, Chengyin Shen, Yaoyao Zhang, Jiarong Wang, Hengjia Wan, Tongwei Zhang, Kun Ma, Wei Lin, Junfeng Wang, Yongxin Pan

**Affiliations:** 1 High Magnetic Field Laboratory, CAS Key Laboratory of High Magnetic Field and Ion Beam Physical Biology, Hefei Institutes of Physical Science, Chinese Academy of Sciences 53040 , Hefei, China; 2 University of Science and Technology of China 12652, Hefei, China; 3 Key Laboratory of Planetary Science and Frontier Technology, Institute of Geology and Geophysics, Chinese Academy of Sciences, Beijing, China; 4 Anhui Province Key Laboratory of Medical Physics and Technology, Institute of Health and Medical Technology, Hefei Institutes of Physical Science, Chinese Academy of Sciences 53040 , Hefei, China; 5 International Magnetobiology Frontier Research Center (iMFRC), Hefei, China; Colorado School of Mines, Golden, Colorado, USA

**Keywords:** magnetobiological effects, 7 T magnetic field, magnetotactic bacteria, biomineralization, denitrification metabolism

## Abstract

High magnetic fields represent an extreme physical condition with growing applications in advanced materials and magnetic resonance technologies. However, the physiological and molecular responses of organisms under such conditions remain poorly characterized. Understanding these responses is essential for elucidating magnetobiological mechanisms and enabling biomedical and engineering innovations. Owing to their intrinsic magnetic sensitivity and well-characterized metabolic pathways, magnetotactic bacteria (MTB) offer an ideal model for investigating biological responses to high magnetic fields. Here, we systematically investigated the impact of a steady-state 7 Tesla (7 T) magnetic field on the growth, magnetosome biomineralization, and nitrogen metabolism of the MTB strain *Magnetospirillum gryphiswaldense* strain MSR-1 (MSR-1). Exposure to the 7 T magnetic field significantly enhanced bacterial proliferation and increased magnetosome number, without altering the average size of individual magnetosome crystals. Transcriptomic analysis demonstrated upregulation of genes encoding the key denitrification enzymes, indicating a pivotal role for nitrogen metabolism in MSR-1 adaptation to the 7 T high magnetic field stress. These transcriptomic results were further supported by proton transfer reaction-mass spectrometry, which confirmed the enhanced denitrification activity under 7 T magnetic field by measuring the intermediate NO and N_2_O gas production. Collectively, our findings provide new insights into the biological effects of high magnetic field on MTB and identify nitrogen metabolism as a key mechanism for MTB adaptation to extreme magnetic environments. These work advances our understanding of microbial magnetic responses and expands the knowledge of magnetobiology.

**IMPORTANCE:**

The physiological impacts of high magnetic fields on microorganisms remain poorly understood. Here, we establish a stable 7 Tesla static magnetic field platform to investigate how *Magnetospirillum gryphiswaldense* MSR-1 responds to extreme magnetic environments. We show that high-field exposure accelerates bacterial growth and increases magnetosome production, coinciding with transcriptional upregulation of key denitrification genes. Using transcriptomics and proton transfer reaction-mass spectrometry, we uncover a metabolic shift toward enhanced redox regulation, suggesting that denitrification plays a central role in magnetic field adaptation. These findings uncover a previously underappreciated connection between magnetic field sensing and metabolic control, providing new insights into how prokaryotes modulate redox homeostasis in extreme environments.

## INTRODUCTION

Magnetic fields are fundamental physical factors in the environment that have important influences on biological systems. Over extensive evolutionary periods, living organisms on earth have evolved specific adaptive mechanisms in response to the Earth’s geomagnetic field (1). In recent decades, there has been growing interest in understanding how various magnetic fields and intensities affect biological responses. For instance, hypogeomagnetic fields have been shown to significantly alter seed germination rates and directional root growth in plants (2); alternating magnetic fields modulate neuronal excitability and nerve signal transmission (3); and gradient magnetic fields influence enzymatic activities and metabolic pathways in plants (4). These findings not only improved our understanding of magnetobiological effects but also offered significant potential for practical applications in medicine, biotechnology, and engineering. One prominent application is the advancement of high-field magnetic resonance imaging (MRI), such as the 7 Tesla (7 T) MRI, which substantially improved spatial resolution and diagnostic accuracy (5). However, the increasing use of high magnetic fields has also raised concerns about their biological effects and potential safety risks. A recent study has demonstrated that static magnetic field (SMF) of 9.4T can alleviate imatinib mesylate-induced toxicity and depression-like behavior in mice (6). Additionally, prolonged exposure to a moderate SMF delayed natural aging processes and ameliorated renal injuries induced by cisplatin chemotherapy in mice (7, 8). Similarly, magnetic fields have been shown to influence microbial physiology, including growth and metabolic activity in *Escherichia coli*, *Staphylococcus aureus*, and *Pseudomonas aeruginosa* (9–12).

Despite these advances, systematic investigations into biological responses under high magnetic field conditions remain limited. The intricate structural and functional networks in living organisms, coupled with the absence of universally recognized magnetosensory system, pose significant challenges in deciphering magnetic field effects (13). Moreover, variations in magnetic field parameters (such as intensity, direction, and exposure duration) further increase experimental complexity. Therefore, selecting appropriate model organisms with simple structures, well-characterized magnetosensory mechanisms, and tractable physiological responses is crucial for mechanistic studies of magnetic effects.

Magnetotactic bacteria (MTB) are ideal model organisms for magnetobiological research due to their intrinsic magnetic sensitivity (14). MTB biomineralize nanoscale magnetic crystals known as magnetosomes, arranged into intracellular chains, allowing cells to orient and navigate along geomagnetic field lines (15–20). This unique arrangement endows MTB with exceptional magnetic sensitivity, amplifying their magnetic response relative to other organisms. Moreover, MTB has relatively simple cellular architectures, clearly defined genetic backgrounds, and well-characterized biomineralization and metabolic regulatory pathways, thus making them particularly suitable for mechanistic investigations of magnetic biological effects (21–23).

Previous research has revealed various MTB responses to low and moderate magnetic field conditions. For example, exposure to low-intensity alternating magnetic fields (e.g., 50 Hz pulsed magnetic field) promotes magnetosome synthesis and maturation in *Magnetospirillum magneticum* strain AMB-1 (AMB-1) (24), while SMF of 1.5 mT stimulates AMB-1 growth but inhibits magnetosome formation through downregulation of genes related to sulfate reduction pathways (25). Similarly, exposure of *Magnetospirillum gryphiswaldense* strain MSR-1 (MSR-1) to moderate SMF (500 mT) enhances magnetosome formation and significantly alters antioxidant enzyme activities (26).

Nevertheless, systematic studies exploring MTB physiological and metabolic adaptations under high magnetic fields (>1 T) remain limited (24–29). High magnetic fields may induce unique cellular and metabolic adaptations. However, their specific impacts on MTB growth, metabolism, and magnetosome biomineralization have not been clearly characterized. This research gap primarily arises from the technical difficulty in maintaining stable, long-term exposure to high magnetic field conditions, which imposes significant constraints on experimental progress.

To address these limitations, we established a stable 7 T SMF experimental platform and employed MSR-1 as a model system to systematically investigate the impact of high magnetic field exposure on bacterial growth, magnetosome biomineralization, and metabolic responses. By integrating molecular biology approaches with proton transfer reaction-mass spectrometry (PTR-MS), we further elucidated the underlying mechanisms of 7 T magnetic field-induced metabolic regulation in MSR-1. Our findings provide new insights into microbial magnetoreception, expand current knowledge in high-field magnetobiology, and provide a foundation for further studies on the interactions between biological systems and high magnetic fields.

## MATERIALS AND METHODS

### 7 T magnetic field treatment

The 7 T magnetic field treatment was conducted using a superconducting magnet system (30) (Model XSMT-10T200, Xi'an Juneng Superconducting Magnet Technology Co., Ltd., Xi'an, China), which includes a magnet body, control and monitoring unit, and a compressor. The center of the magnet body can generate a vertical magnetic field with a strength of 7 T, with an excitation current range of 0–116 A and an excitation speed of 0.04 A/s. In addition, the experiment was also equipped with a water-bath thermostatic system (Shanghai Xiren Scientific Instruments Co., Ltd., Shanghai, China) to ensure uniform temperature control across the sample area. The rest of the design and setup were self-completed. For a 7 T magnetic field treatment, samples were placed in the center of the coil with the magnetic field strength set to 7 T, and then incubated in the dark at room temperature for 18 hours.

### Measurement of MSR-1 growth and magnetic sensing ability

According to previously described methods (31), the MSR-1 strain was cultured at 30°C in LA-2 liquid medium, which consisted of the following components per liter: 2 mL of 50% sodium lactate solution, 0.1 g potassium dihydrogen phosphate, 0.15 g magnesium sulfate heptahydrate, 2.38 g hepes, 0.34 g sodium nitrate, 0.1 g yeast extract, 3 g peptone, 0.05 g sodium thioglycolate, 0.5 mL mineral element mixture, and 2 mL of 0.01 M ferric citrate. All reagents were obtained from Shanghai Shengong Biotechnology Services Co., Ltd., Shanghai, China.

To obtain complete growth and magnetic sensing ability (*C*_mag_) curves for MSR-1, the strain was first recovered from 25% glycerol and then cultured without ferric citrate in a shaker (100 rpm, 30°C) (Model ZWY-100H, Shanghai Zhicheng Analytical Instrument Manufacturing Co., Ltd., Shanghai, China) for 20 hours until reaching an optical density at 565 nm (OD_565_) of about 0.9 (near the stationary phase), with an original *C*_mag_ value of 0 (without magnetosomes), and lastly transferred to static conditions with the addition of ferric citrate as previously described (27). All samples were inoculated with an initial OD_565_ of about 0.03, with the bacterial suspension (80 mL) filling most of the bottle volume (100 mL) to facilitate optimal growth conditions for MSR-1 (32). Three biological replicates were prepared for each treatment, yielding a total of six bottles. Of these, three bottles were exposed to the 7 T magnetic field, while the other three bottles served as controls under the geomagnetic field. Measurements were performed at designated time points throughout the 18 hour experimental period. At each time point, the bottles were manually mixed, and 500 µL of culture was withdrawn using sterile syringes for OD and *C*_mag_ measurements. OD_565_ and *C*_mag_ values were recorded every 2 hours using a 721 visible spectrophotometer (Shanghai Yuke Instrumentation Co., Ltd., Shanghai, China) to construct time-resolved growth and *C*_mag_ curves.

### Magnetic measurement and electron microscopy analysis

MSR-1 cultures (80 mL) collected at 13 and 18 hours were centrifuged at 3,000 rpm for 10 minutes to pellet the cells, followed by freeze-drying for 12 hours. Magnetic properties, including hysteresis loops, first-order reversal curves (FORC), and saturated isothermal remanent magnetization, were measured at room temperature using a VSM3900 magnetometer (Princeton Measurements Corporation, USA) with a sensitivity of 5.0 × 10^−10^ Am^2^ (33). A total of 120 FORC curves were recorded with an applied field step of 0.721 mT and an averaging time of 500 ms (34). FORC diagrams were processed using FORCinel (version 1.18) with a smoothing factor of 3 (35).

For transmission electron microscopy (TEM) analysis, 20 µL 100× concentrated MSR-1 culture was dropped on a copper grid (Beijing Zhongjing Keyi Technology Co., Ltd., Beijing, China). TEM images were acquired using a JEOL JEM-2100 instrument operated at 200 kV. Crystal sizes were manually measured using ImageJ (version 1.51i) (36), and then statistically analyzed using GraphPad Prism (version 9.5.1) (37).

### Transcriptomic analysis and RT-qPCR

MSR-1 cultures incubated for 13 hours were harvested for transcriptomic analysis and reverse transcription quantitative PCR (RT-qPCR). Total RNA was extracted using the TransZol Up Plus RNA Kit, and RNA purity and concentration were determined with a spectrophotometer. The transcriptomic sequencing was performed by Shanghai Majorbio Bio-Pharm Technology Co., Ltd., and subsequent bioinformatic analyses were conducted on the company’s online analysis platform. Complementary DNA (cDNA) was synthesized from the extracted RNA using the TIANGEN FastKing cDNA First-Strand Synthesis Kit. RT-qPCR was performed using the SuperReal PreMix Plus (SYBR Green) Kit on a LightCycler 96 instrument (Roche, Switzerland). Relative gene expression levels were determined using the 2^−ΔΔCt^ method and normalized to rpoC as the reference gene. The experiment was conducted in triplicate. Primers for each gene were synthesized by Shanghai Shengong Biotechnology Services Co., Ltd., Shanghai, China, and are listed in the Table S1. The experimental data were statistically analyzed using GraphPad Prism (version 9.5.1) (37).

### Gas detection in solid media and PTR-MS

To prepare the solid medium, 0.3% agar (Shanghai Shengong Biotechnology Services Co., Ltd., Shanghai, China) was added to the standard culture medium. MSR-1 cells were inoculated and mixed into the medium while it was semi-solid (38). Once the medium had fully cooled and solidified, bubble formation was monitored under two conditions (7 T magnetic field or geomagnetic field). Ten parallel samples were prepared and analyzed for each condition.

For PTR-MS analysis, MSR-1 was cultured in a liquid medium with the culture volume occupying around one-quarter of the total volume of the bottle, allowing sufficient headspace for gas production and collection. Three biological replicates were prepared for the experiment. Gas composition and concentration were analyzed using a PTR-MS system manufactured by Chengyin Shen’s laboratory (39). This system consisted of a hollow cathode discharge ion source, a focusing quadrupole ion funnel drift tube, an ion-transfer lens assembly, a quadrupole mass spectrometer, and a secondary electron multiplier detector. High-density and high-purity hydrated hydrogen ions generated by the ion source entered the drift tube, where they collided with the sample gas and ionized it. Subsequently, the ionized gas passed through a small hole at the end of the drift tube and entered the mass spectrometer for detection. Qualitative and quantitative analyses of the gas were performed based on the data graph generated by the detector. The experimental data were statistically analyzed using Origin (version 7.0) (40).

### Quantification and statistical analysis

For data sets related to gas bubble production and number and size of magnetosomes, they were first tested for normality using the Shapiro-Wilk test. A *P*-value greater than 0.05 indicated a normal distribution. For comparisons between two groups, significance analysis was conducted based on the distribution of the data. If both groups followed a normal distribution, two-sided unpaired Student’s *t*-tests were conducted. If either group did not satisfy the normal distribution, a two-tailed unpaired Mann-Whitney *U*-test was employed.

For the rest data, including OD_565_, *C*_mag_, doubling time, RT-qPCR, and production of NO and N_2_O, two-sided unpaired Student’s *t*-tests were conducted.

The statistical analyses were performed using GraphPad Prism (version 9.5.1) (37) and SPSS (version 31.0.0.0) (41). A *P* < 0.05 was considered statistically significant. *P < 0.05, **P < 0.01, ***P < 0.001, ****P < 0.0001; n.s., not statistically significant.

## RESULTS

### Culture and growth of MSR-1 under the 7 T magnetic field

**Fig 1:**
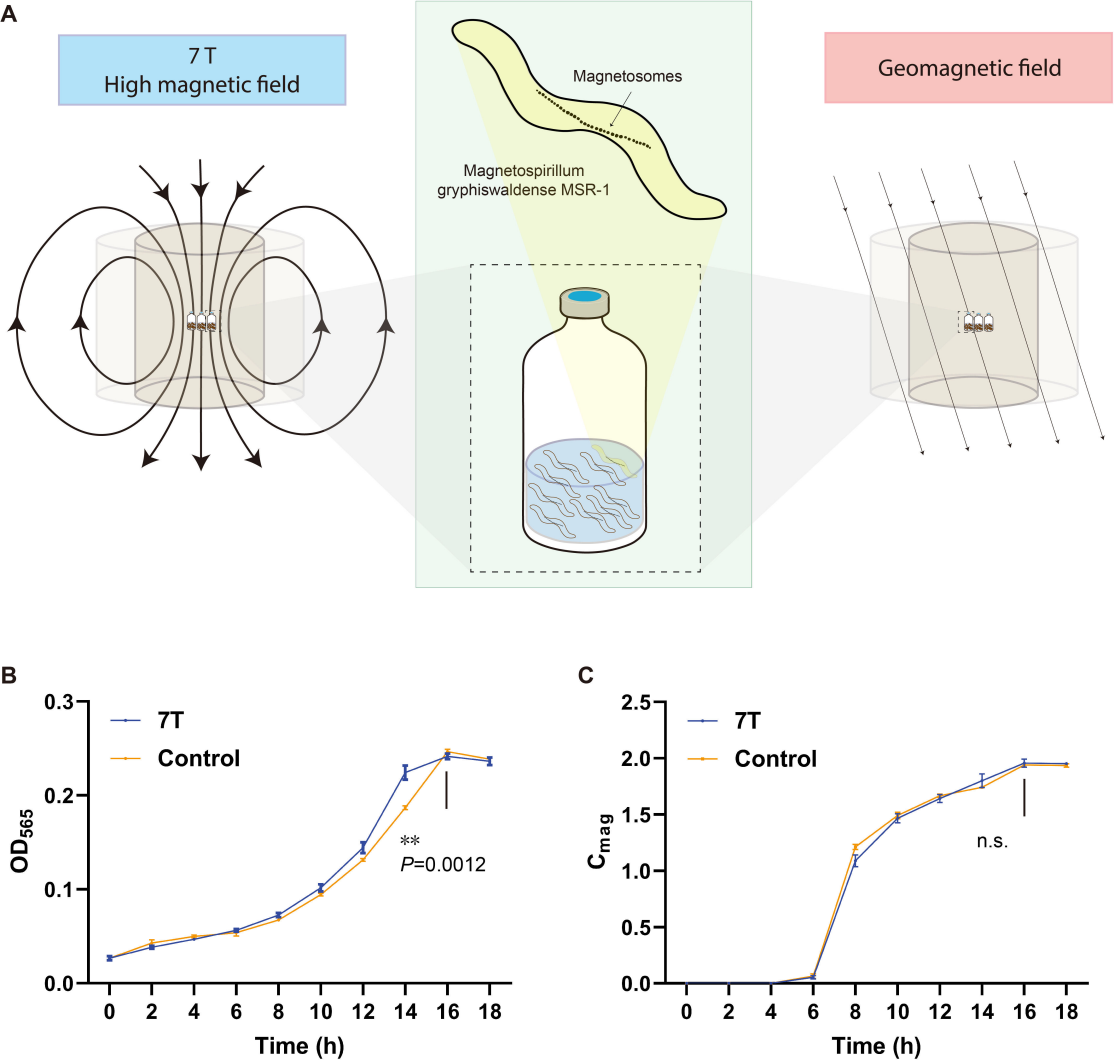
Enhanced growth of MSR-1 under a 7 T magnetic field. (**A**) Schematic diagram of the experimental setup. (**B and C**) Time-course monitoring of OD_565_ and *C*_mag_ of MSR-1 over 18 hours under 7 T magnetic field and geomagnetic field (control) conditions. The number of samples in each group was three (*n* = 3). *P*-values were calculated by the two-sided unpaired Student’s *t*-tests (**B and C**). **P < 0.01; n.s., not statistically significant.

To investigate the magnetobiological effects of high magnetic fields, we first established a stable magnetic field environment. As shown in Fig. S1 and S2, the bottles (*n* = 3) containing MSR-1 cultures were placed in a uniform 7 T magnetic field generated by a superconducting magnet, while the control bottles (*n* = 3) were placed in the geomagnetic field (61.53 μT) (Fig. 1A). The difference in magnetic field strength between the experimental and control groups was approximately 113,766-fold, proving a pronounced contrast to assess changes in magnetic field-induced biological effects (42).

Growth and magnetic sensing ability of MSR-1 were monitored over an 18 hour period. The values of OD_565_ (Fig. 1B) indicated that differences in growth emerged at the 14 hours, with the 7 T-exposed cultures entering the stationary phase earlier than the control group. The doubling time decreased from 3.93 hours in the control to 3.15 hours under the 7 T magnetic field condition, representing a 24.96% increase in growth rate (Fig. S3). This acceleration indicates that high magnetic field exposure can improve fermentation efficiency by reducing cultivation time and resource input. Meanwhile, the 7 T magnetic field did not significantly affect the magnetic sensing ability of MSR-1, which retained its original biological characteristics (Fig. 1C), indicating that the enhanced growth occurred without compromising MSR-1’s magnetic sensing ability.

### Analysis of the 7 T magnetic field on the magnetic properties of MSR-1

To determine whether the unaltered magnetic sensing ability of MSR-1 under the 7 T magnetic field was a result of complete lack of magnetic influence or due to compensatory changes in magnetic properties, we conducted a detailed analysis of its magnetic characteristics. Based on the growth curve data, we selected samples from 13 hours (when differences emerged) and 18 hours (the end of the experiment) for magnetic analyses.

**Fig 2:**
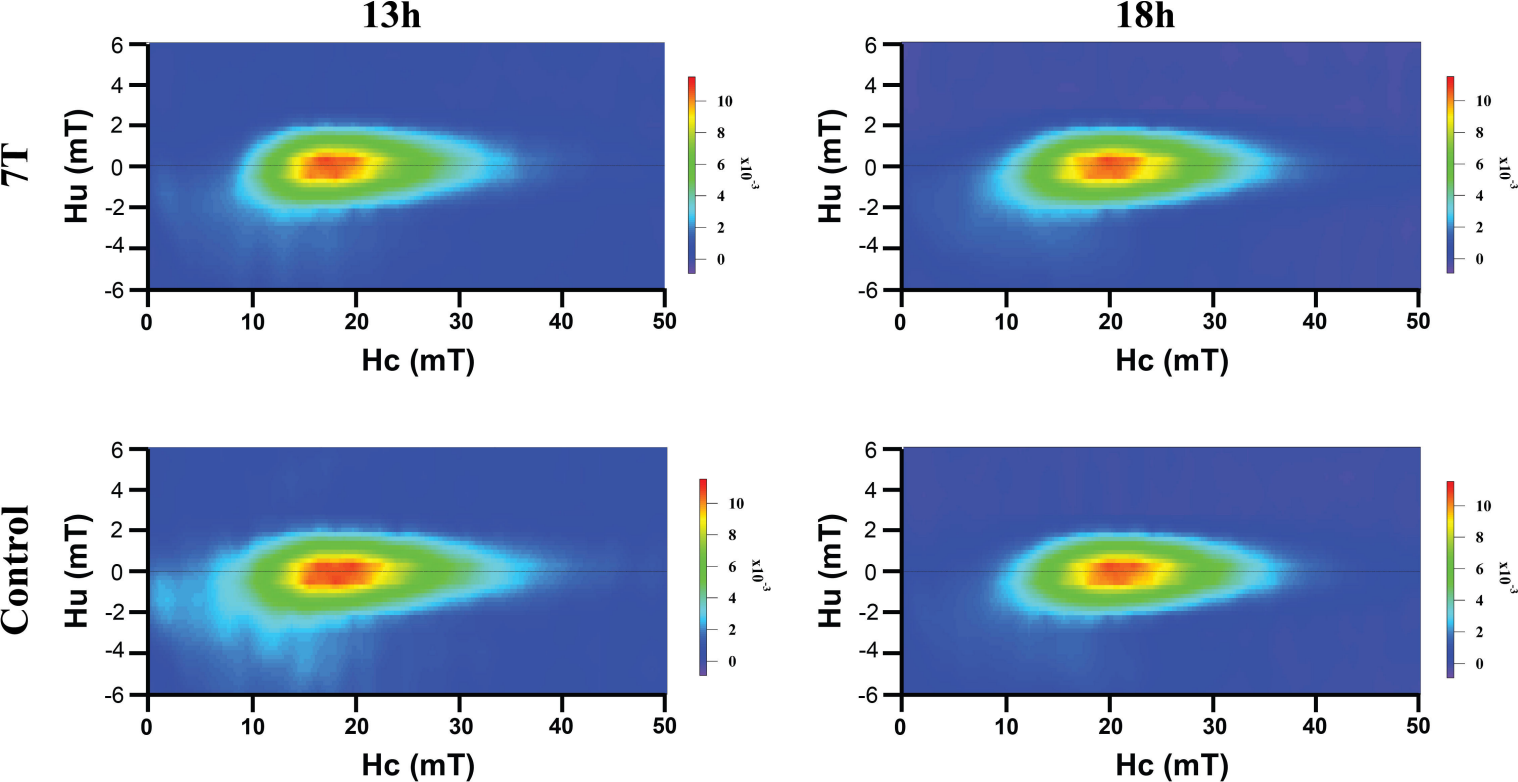
Minimal changes in magnetic properties under 7 T magnetic field treatment. FORC diagrams of MSR-1 cultured under 7 T magnetic field and geomagnetic field (control) conditions at different treatment time points.

FORC diagrams (Fig. 2) revealed complete and closed contours in all samples. These contours were narrowly distributed along the *y*-axis but widely distributed along the *x*-axis, forming elongated elliptical shapes with the long axis aligned horizontally. Referring to the relevant analysis (43), this pattern indicated that the samples were predominantly composed of single-domain magnetite with weak magnetic interactions.

Afterward, in order to get more detailed magnetic properties, we conducted tests with SQUID. Compared to the control samples cultured under geomagnetic field, only minor differences were observed in key magnetic parameters, including coercivity, remanent coercivity, saturation remanence, saturation magnetization, and the Mrs/Ms ratio (Table 1). These results are consistent with the minimal change in magnetic sensing ability mentioned above. However, the 18 h-7 T sample may exhibit subtle changes in magnetic properties, with the particles possibly tending toward lower coercivity, remanent coercivity, saturation remanence, and the Mrs/Ms ratio. This implied that prolonged exposure to the 7 T magnetic field may lead to structural modifications in magnetosomes. As suggested by the related study of MTB, the metabolites under many extreme conditions may be detrimental to biomineralization (44). Therefore, this phenomenon may be due to adaptations to prolonged exposure to the 7 T magnetic field, aiming to better reduce the interference of the external environment on internal energy conversion or other related biological activities.

**TABLE 1:** Magnetic properties of MSR-1 under different magnetic field treatments^*a*^

Samples	Coercivity (mT)	Remanent coercivity (mT)	Saturation remanence (mAm^2^/g_[Dry weight]_)	Saturation magnetization (mAm^2^/g_[Dry weight]_)	Mrs/Ms
13 h-control	11.79	19.36	0.26	0.72	0.36
13 h-7 T	12.69	19.42	0.26	0.73	0.36
18 h-control	15.96	21.34	0.26	0.58	0.45
18 h-7 T	14.10	20.74	0.23	0.59	0.39

^*a*^ The dry weights of MSR-1 were 4.1 mg (13 h-control), 3.9 mg (13 h-7 T), 5.2 mg (18 h-control), and 5.9 mg (18 h-7 T).

**Fig 3:**
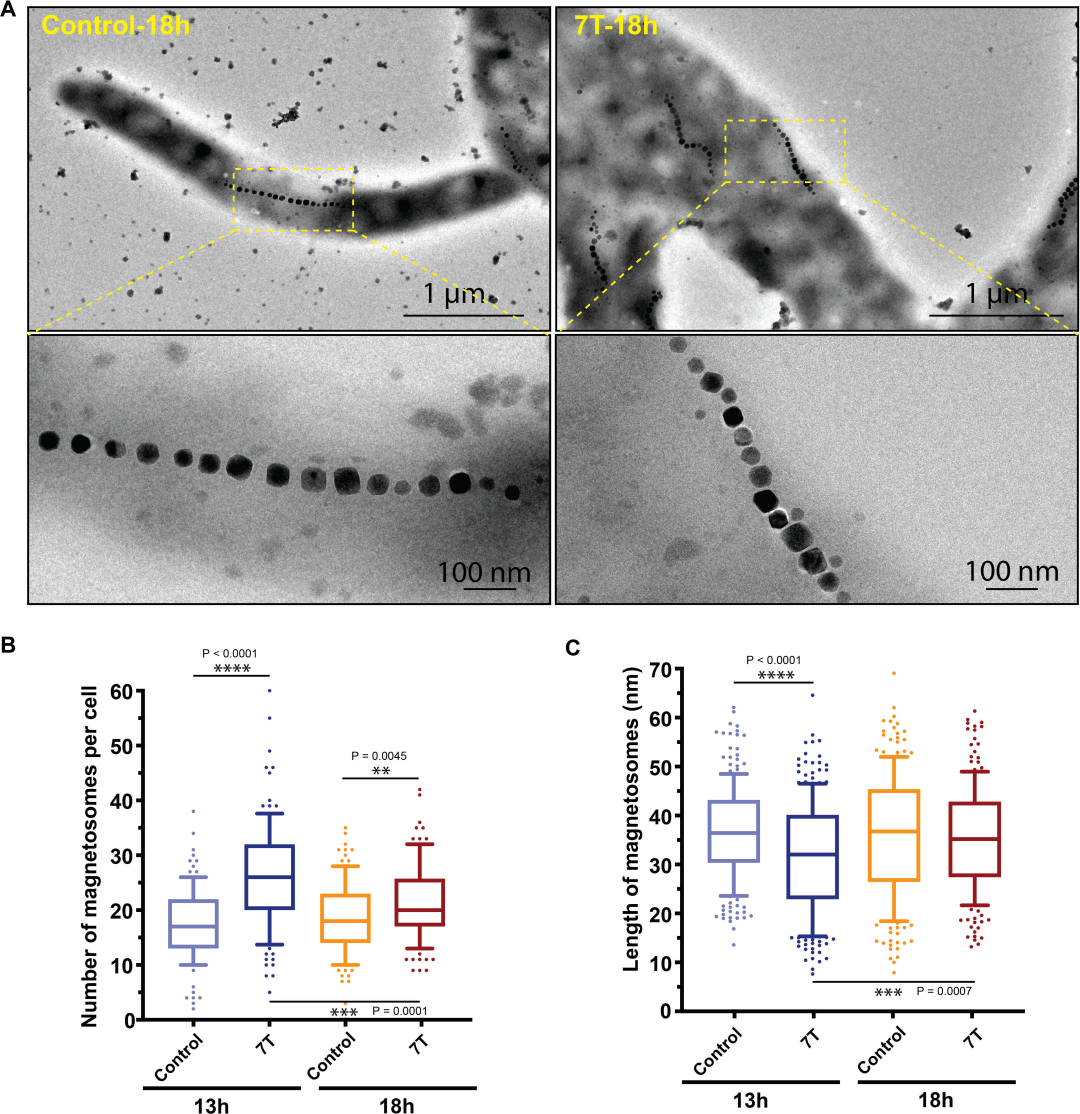
Effects of the 7 T magnetic field on magnetosome biomineralization in MSR-1. (**A**) TEM images and locally enlarged views of cells cultured under 7 T magnetic field and geomagnetic field conditions. (**B and C**) Statistical analyses of the number of magnetosomes per cell (**B**) and the length of magnetosomes (**C**) based on TEM observations. The numbers of bacterial cells counted in panel B, from left to right, were 105, 101, 103, and 104, respectively. The numbers of magnetosomes counted in panel C, from left to right, were 221, 259, 222, and 212, respectively. *P*-values were calculated using the two-tailed unpaired Mann-Whitney *U*-test. **P < 0.01, ***P < 0.001, ****P < 0.0001.

To further assess the effects of the 7 T magnetic field on magnetosome biomineralization in MSR-1, TEM was used to visualize the magnetosome chains in MSR-1 (Fig. 3A). Quantitative analysis was performed on numerous TEM images to statistically evaluate the number of magnetosomes per cell (Fig. 3B). The results showed a significant increase in magnetosome number at both 13 hours and 18 hours under 7 T magnetic field exposure compared to the control. Meanwhile, the number of magnetosomes in the log phase was prominently higher than that in the stationary phase, indicating that the effect of the 7 T magnetic field on magnetosome formation primarily occurs during active cell growth. Additionally, the length of magnetosomes was measured, and the result revealed that the magnetosome particles became notably smaller in the log phase and then recovered to normal levels in the stationary phase. A previous study reported a certain elemental balance during growth of MSR-1 (45). Therefore, this observation may be due to alterations in the balance between the quantity and size of magnetosomes influenced by the 7 T magnetic field during biomineralization. Together with the data from Table 1, these findings indicate that this balance may still exist during the log phase, but may be disrupted in the stationary phase under prolonged high magnetic field exposure.

### Effects of the 7 T magnetic field on the transcriptome of MSR-1

**Fig 4:**
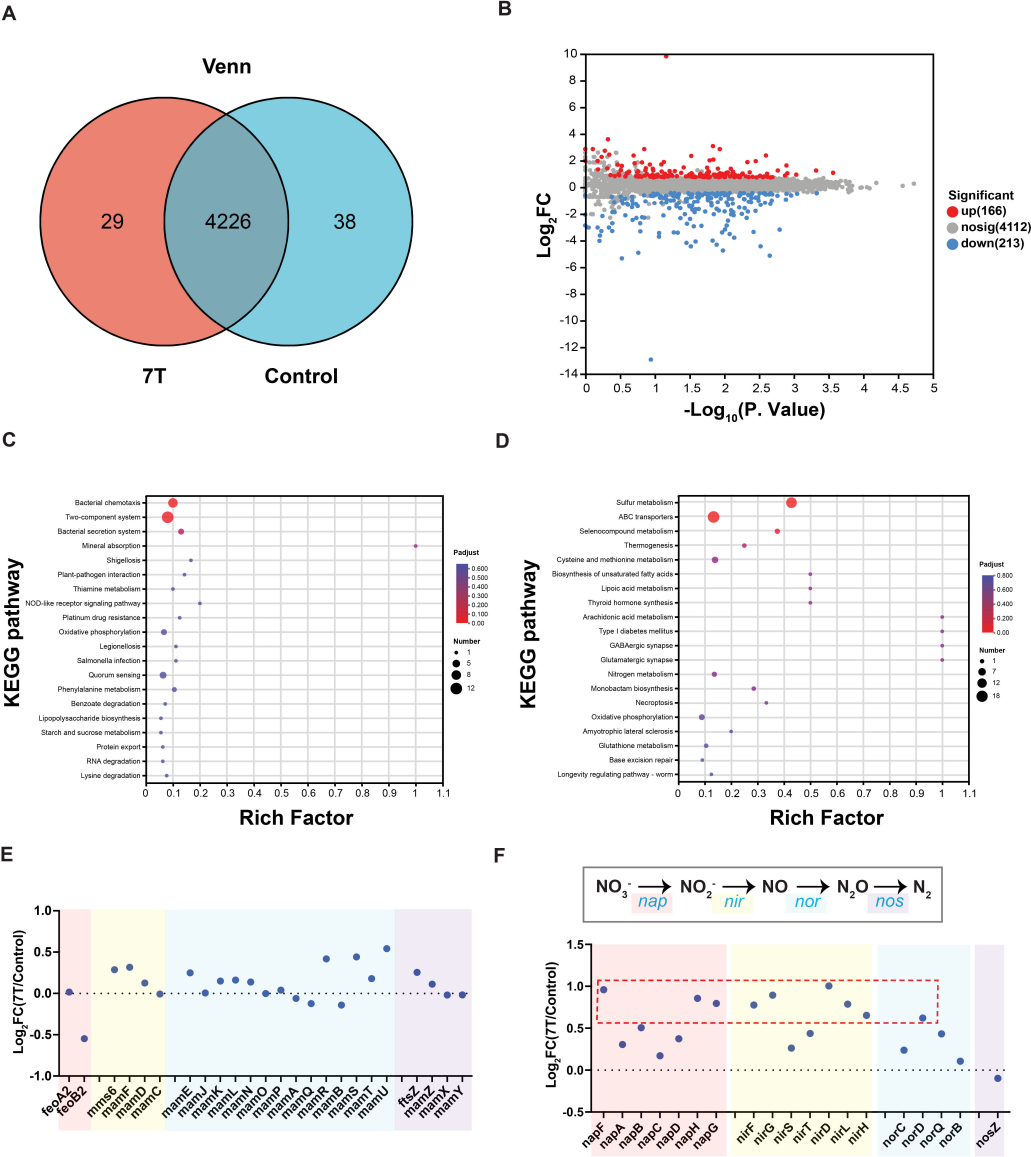
Transcriptomic analysis of MSR-1 under 7 T magnetic field and geomagnetic field (control) conditions. (**A**) Venn diagram showing the gene expression difference under the two magnetic field treatments. (**B**) Volcano plot displaying significantly upregulated and downregulated genes. (**C and D**) KEGG pathway enrichment analysis of upregulated pathways (**C**) and downregulated pathways (**D**). (**E**) Statistical analysis of gene expression related to magnetosome formation. (**F**) Overview of the denitrification pathway (up panel) in nitrogen metabolism, with statistical analysis of expression levels for four key enzyme types (lower panel). Log_2_FC: the fold change in the 7 T group relative to the control group, then taken as the logarithm to base 2.

After confirming that the magnetic properties of MSR-1 were affected under the 7 T magnetic field, we further conducted transcriptomic analysis on samples collected at 13 hours, especially the gene expression related to magnetic properties. As shown in the Venn diagram in Fig. 4A, the majority of genes (4,226) were expressed in both conditions, while a small subset was unique to either the 7 T magnetic field treatment (29 genes) or the control group (38 genes). Most of these condition-specific genes encode hypothetical proteins with unknown functions, indicating they may play a critical role in the high magnetic response. Furthermore, differential expression analysis (Fig. 4B; Table S2) revealed 166 significantly upregulated and 213 downregulated genes (*P* < 0.05) under 7 T exposure.

KEGG pathway enrichment of the upregulated genes (Fig. 4C) indicated activation of pathways associated with chemotaxis, two-component system, secretion system, and mineral absorption. Based on previous studies of biomineralization processes (14, 16), these upregulated pathways were closely associated with magnetosome synthesis. In contrast, the downregulated genes were predominantly enriched in metabolic pathways, including sulfur and nitrogen metabolism, cysteine and methionine metabolism, and ABC transporters (Fig. 4D), indicating a broader metabolic effect in response to high magnetic field exposure.

As suggested by previous studies (19, 46), these gene expression changes likely reflect a complex interplay of regulatory networks, with some changes resulting directly or indirectly from the 7 T magnetic field exposure, making it difficult to pinpoint the upstream and downstream modification flux. To clarify the underlying mechanism, we first focused on the genes and metabolic pathways directly related to magnetosome biomineralization. Analysis of the magnetosome gene cluster revealed minimal transcriptional changes under 7 T conditions (Fig. 4E), indicating that the genes for magnetosome formation were largely unaffected. Inspired by previous reports of a close connection between nitrogen metabolism for energy production and biomineralization or magnetic field (38, 47–49), we next focused on the related gene expression of nitrogen metabolism, especially in denitrification. Denitrification is an anaerobic respiratory process in which nitrate (NO₃^-^) serves as an electron acceptor and is sequentially reduced to nitrite (NO₂^-^), nitric oxide (NO), nitrous oxide (N_2_O), and finally dinitrogen gas (N_2_), facilitated by four key enzyme systems: Nap, Nir, Nor, and Nos (50). Under 7 T magnetic field exposure, the expression levels of genes encoding all four enzyme types were upregulated (Fig. 4F), indicating that enhanced denitrification may play an essential role in the cellular adaptation and magnetosome biomineralization of MSR-1 in response to the 7 T high magnetic field.

### Regulation of denitrification in MSR-1 under the 7 T magnetic field

**Fig 5:**
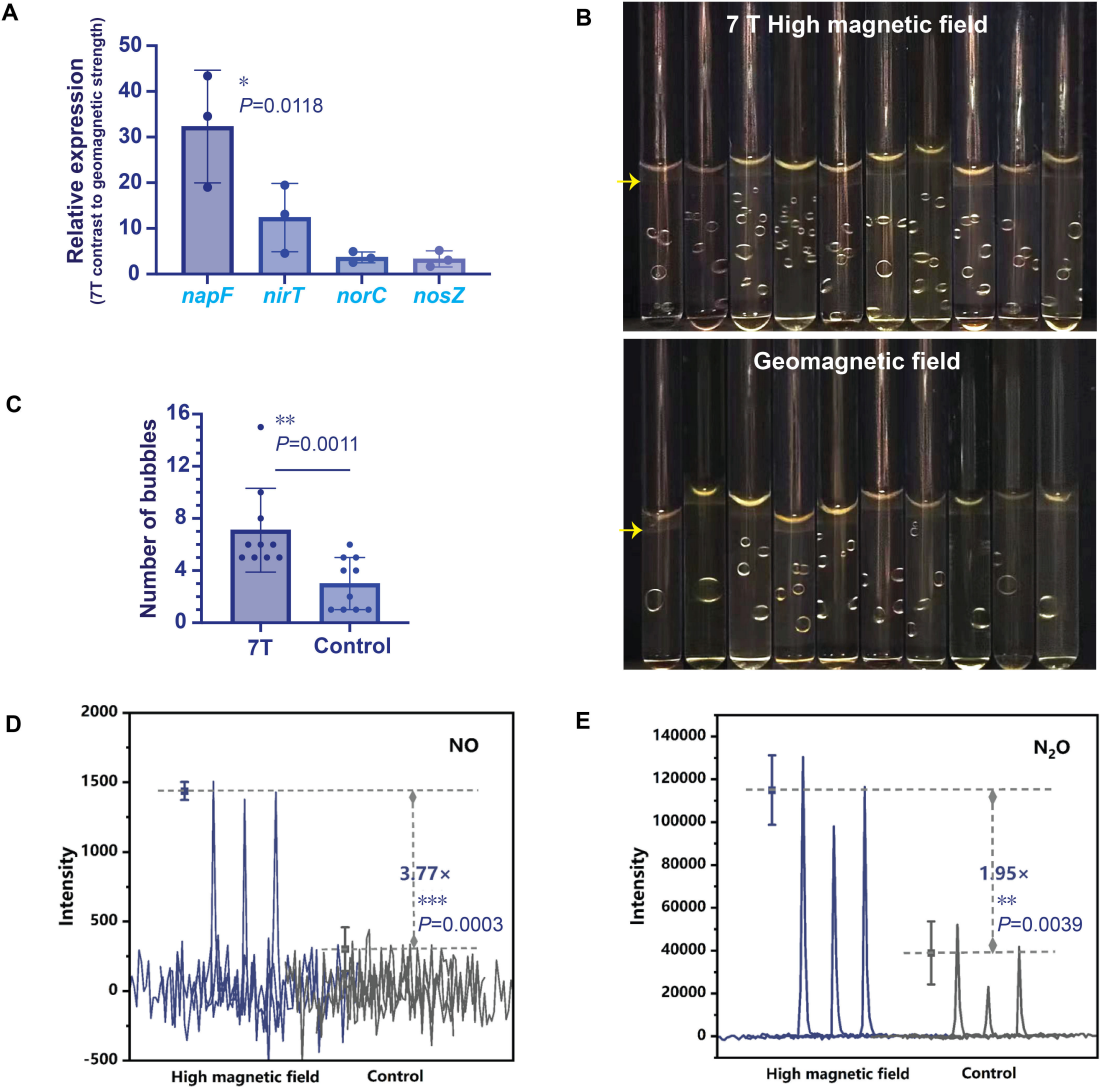
Increased expression of denitrification-related enzymes and intermediate products under exposure to a 7 T magnetic field. (**A**) RT-qPCR validation of the upregulated expression of four denitrification-related enzymes. (**B and C**) Monitoring and statistical analyses of gas bubble production in the MSR-1 gas production experiment. (**D and E**) Comparison of NO (**D**) and N_2_O (**E**) production between 7 T magnetic field and geomagnetic field treatments, as detected by PTR-MS. The numbers of samples in each group were three for panels A, D, and E (*n* = 3), and 10 for panels B and C (*n* = 10). *P*-values were calculated using the two-sided unpaired Student’s *t*-tests (**A, D, and E**) and the two-tailed unpaired Mann-Whitney *U*-test (**C**). *P < 0.05, **P < 0.01, ***P < 0.001.

To further validate the transcriptomic findings, we experimentally examined the role of denitrification in MSR-1 under a 7 T magnetic field. Firstly, we conducted RT-qPCR to check the expression of four key denitrification enzymes. As shown in Fig. 5A, expression levels of all four enzymes were upregulated, with *napF* exhibiting particularly strong increases. These results indicate that the 7 T magnetic field likely exerted primary influence by modulating the initial stages of the denitrification pathway, and then the accumulation of early intermediate products may substantially promote the overall denitrification process (45).

Given that denitrification involves the production of various gases, we next aimed to further validate the gene expression data by examining gas production. We initially cultivated MSR-1 in solid medium to retain produced gases and prevent their escape. Visual inspection revealed that samples exposed to the 7 T magnetic field formed significantly more gas bubbles than the control group (Fig. 5B and C), indicating enhanced gas production. This observation is consistent with previous findings by Yingjie Li et al. in Δ*nirS* mutant, where disruption of denitrification pathways affected gas bubbles formation (38). To quantify the effect, we used PTR-MS (Fig. S4) to detect NO and N_2_O, two measurable intermediate products of denitrification. The 7 T-treated samples showed a 3.77-fold increase in NO (Fig. 5D) and a 1.95-fold increase in N_2_O (Fig. 5E) compared to controls. Taken together, these results confirm that exposure to a 7 T magnetic field results in enhanced expression of denitrification-related enzymes and increased accumulation of corresponding gaseous products in MSR-1.

## DISCUSSION

Understanding the biological effects of high magnetic fields is essential for elucidating magnetoresponsive mechanisms in living systems, ensuring the biosafety of high magnetic field technologies, and advancing applications in medicine and bioengineering (51, 52). As prototypical unicellular magnetosensitive organisms, MTB serve as an ideal model for investigating magnetobiological phenomena. Moreover, numerous experiments involving varying magnetic field gradients have shown that applying high magnetic fields can amplify subtle magnetobiological effects in most cases, thereby facilitating their observation and mechanistic characterization (42, 53, 54). In this study, we employed MSR-1 to systematically assess its growth dynamics, biomineralization, and gene expression under exposure to the 7 T static magnetic field. Our results reveal that 7 T high magnetic field exposure modulates MSR-1 growth and magnetosome biomineralization via regulation of denitrification metabolism.

Several studies have been performed previously to investigate the effects of different SMF conditions on the biomineralization of MTB. Exposure of the AMB-1 strain to the 1.5 mT magnetic field after 20 hours resulted in a higher OD compared to the geomagnetic field condition, whereas the *C*_mag_ value decreased. Meanwhile, no significant change was observed in the number of intracellular magnetosomes, but the magnetosome size was reduced, accompanied by a decline in saturation magnetization (25). Another study further demonstrated that under the 0.2T magnetic field, AMB-1 exhibited impaired growth, along with an increased *C*_mag_ value in whole, and a higher number of magnetosomes, an elevated iron consumption with exposure after 16 hours (27). In contrast, the results obtained in this study after 13 hours of exposure to the 7 T magnetic field showed some differences. Compared with the geomagnetic control group, MSR-1 displayed a significant increase in OD, while the *C*_mag_ value and magnetosome properties remained unchanged, although the number of magnetosomes increased markedly and the length changed inversely. Notably, as time progresses to the stationary phase with exposure to the 7 T magnetic field after 18 hours, compared with the geomagnetic control group, the differentiation in magnetosome length disappeared while their quantity continued to remain at a notably increasing level.

The biomineralization of magnetosomes is closely associated with the metabolic processes of MTB (55, 56). By performing transcriptomic analysis comparing the 7 T magnetic field group with the geomagnetic field control, KEGG enrichment revealed downregulation of genes involved in the sulfur metabolism pathway under the 7 T magnetic field condition. This finding is consistent with the report of Chen et al., where exposure of AMB-1 to the 1.5 mT SMF led to increased intracellular reactive oxygen species (ROS), which subsequently downregulated the expression of adenylyl-sulfate kinase *CysC* in the sulfate reduction pathway, thereby affecting bacterial growth and magnetosome biomineralization. It has been proposed that magnetosome particles may exhibit intrinsic nanozyme-like activity, serving as a potential mechanism for ROS scavenging (57, 58). Furthermore, high magnetic field exposure has also been reported to principally increase intracellular ROS generation (59). Based on these findings, we speculate that to mitigate the associated oxidative stress, MTB may accelerate magnetosome biomineralization as an adaptive mechanism to efficiently eliminate accumulated ROS.

Denitrification is a fundamental biochemical process in MSR-1 that converts NO₃⁻ to N₂ and harvests energy. Additionally, numerous studies had confirmed a close relationship between denitrification and biomineralization (45, 47, 49, 50, 60). The denitrification pathway involves numerous electron transfer components, such as the electron donor NADH, electron carrier iron-sulfur clusters ([4Fe–4S]), molybdenum cofactor, and others (61), which may be possible targeted points influenced by magnetic fields (62–64). One study reported that the 0.6 mT magnetic field can impact denitrification by affecting metalloprotein expression in denitrifying microbial communities (65). Furthermore, nitrogen consumption had been shown to increase during the initial stages, mediating the formation of magnetosome vesicles and providing energy (45). We also demonstrated that the 7 T magnetic field primarily affected the early part of the denitrification process. Therefore, the accelerated growth during the log phase may be indirectly caused by the magnetic field’s influence on denitrification. During magnetosome synthesis, NO acts as a signaling molecule regulating magnetosome formation (47), and iron reduction is also linked to nitrate reduction (49). Disrupting the denitrification pathway can further affect bacterial growth and magnetosome formation (50). Moreover, effectively controlling the expression of denitrification genes is beneficial for maintaining redox balance during the mineralization process, as these key enzymes are involved in the biomineralization of magnetosomes (60). Therefore, denitrification may serve as a connecting link between magnetic fields and biomineralization.

Our study not only identifies potential biochemical targets influenced by high magnetic fields but also provides novel insights into practical applications of MTB. MSR-1, characterized by rapid growth rates and high saturation OD, is widely employed in MTB fermentation processes, producing valuable products relevant to biomedicine, chemical synthesis, and industry (66, 67). We demonstrated that exposure to the 7 T magnetic field significantly accelerated MSR-1 growth by approximately 24.96% during the log phase, highlighting the potential of high magnetic fields to substantially improve fermentation efficiency.

Despite identifying a potential biochemical pathway influenced by the 7 T magnetic field in MSR-1, the precise molecular targets and regulatory mechanisms remain unclear. Therefore, future research could focus on identifying magneto-responsive molecular targets and elucidating molecular-level magnetobiological effects involving key enzymes, such as nitrate reductase. Additionally, we primarily investigated the denitrification related to biomineralization in MSR-1, without exploring others more. Future studies could broaden the scope to analyze additional MTB strains and other metabolic pathways, providing a comprehensive understanding of the universal mechanisms about MTB adaptation and biological responses under high magnetic fields.

## SUPPLEMENTAL MATERIAL

Supplemental material aem.01069-25-s0001.pdf Fig. S1 to S4; Tables S1 and S2.

ASM does not own the copyrights to Supplemental Material that may be linked to, or accessed through, an article. The authors have granted ASM a non-exclusive, world-wide license to publish the Supplemental Material files. Please contact the corresponding author directly for reuse.
