# Supplementary material for: Influence of a 7 T magnetic field on growth, biomineralization, and denitrification metabolism in Magnetospirillum gryphiswaldense MSR-1
Source: Appl Environ Microbiol. 2025 Sep 17;91(10):e01069-25. doi: 10.1128/aem.01069-25 (PMC12542674; doi:10.1128/aem.01069-25)
Supplement: Supplemental material — Fig. S1 to S4; Tables S1 and S2. [file aem.01069-25-s0001.pdf]

Supplementary information

Influence of a 7 T Magnetic Field on Growth, Biomineralization, and Denitrification Metabolism in *Magnetospirillum gryphiswaldense* MSR-1

Jing Zhang[#1,2], Juan Wan[#3], Chengyin Shen[4], Yaoyao Zhang[1,2], Jiarong Wang[1], Hengjia Wan[1,2], Tongwei Zhang*[3], Kun Ma*[1,5], Wei Lin[3], Junfeng Wang[1,2,5], Yongxin Pan[3]

1. High Magnetic Field Laboratory, CAS Key Laboratory of High Magnetic Field and Ion Beam Physical Biology, Hefei Institutes of Physical Science, Chinese Academy of Sciences, Hefei 230031, P.R. China.

2. University of Science and Technology of China, Hefei 230026, P.R. China.

3. Key Laboratory of Planetary Science and Frontier Technology, Institute of Geology and Geophysics, Chinese Academy of Sciences, Beijing 100029, China.

4. Anhui Province Key Laboratory of Medical Physics and Technology, Institute of Health and Medical Technology, Hefei Institutes of Physical Science, Chinese Academy of Sciences, Hefei 230031, P.R. China

5. International Magnetobiology Frontier Research Center (iMFRC), Hefei 230031, P.R.China.

*Corresponding authors: ztw@mail.iggcas.ac.cn (Tongwei Zhang);
makun@hmfl.ac.cn (Kun Ma)

Figure S1

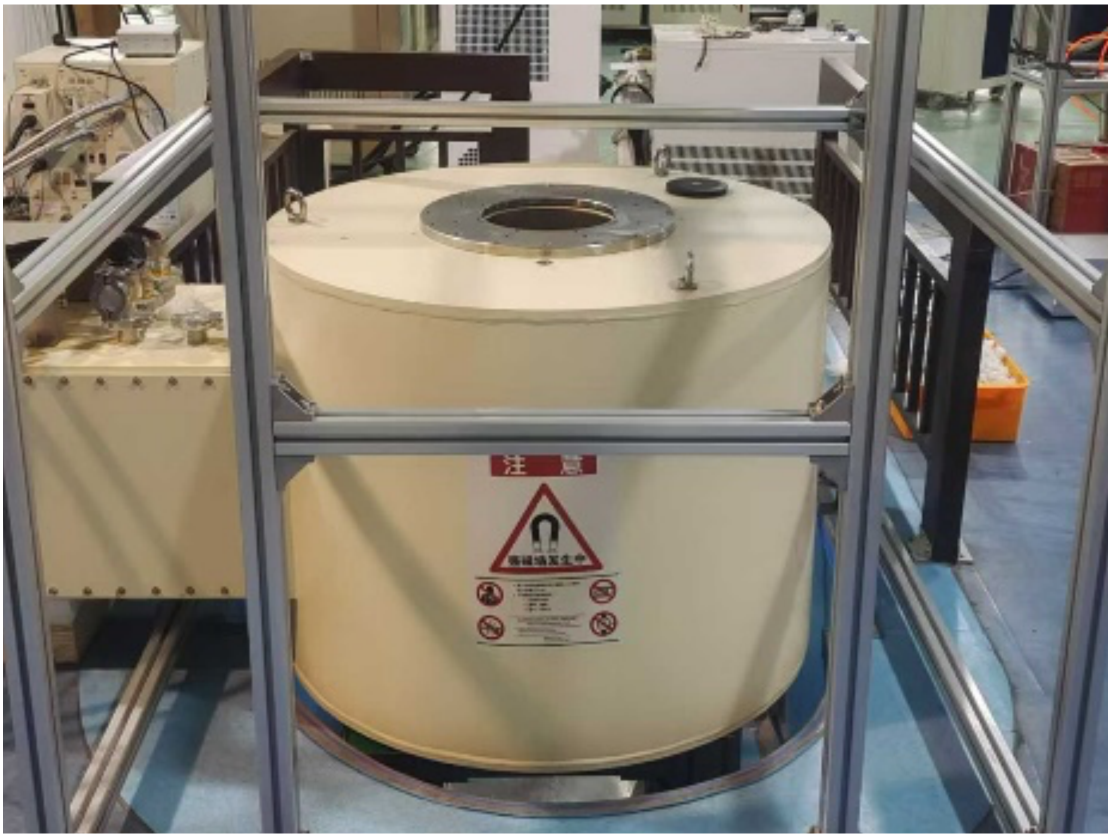

Figure S1. Photograph of the magnetic equipment, which is composed of a superconducting magnet body, a control and monitoring system, a compressor, and other associated components.

Figure S2

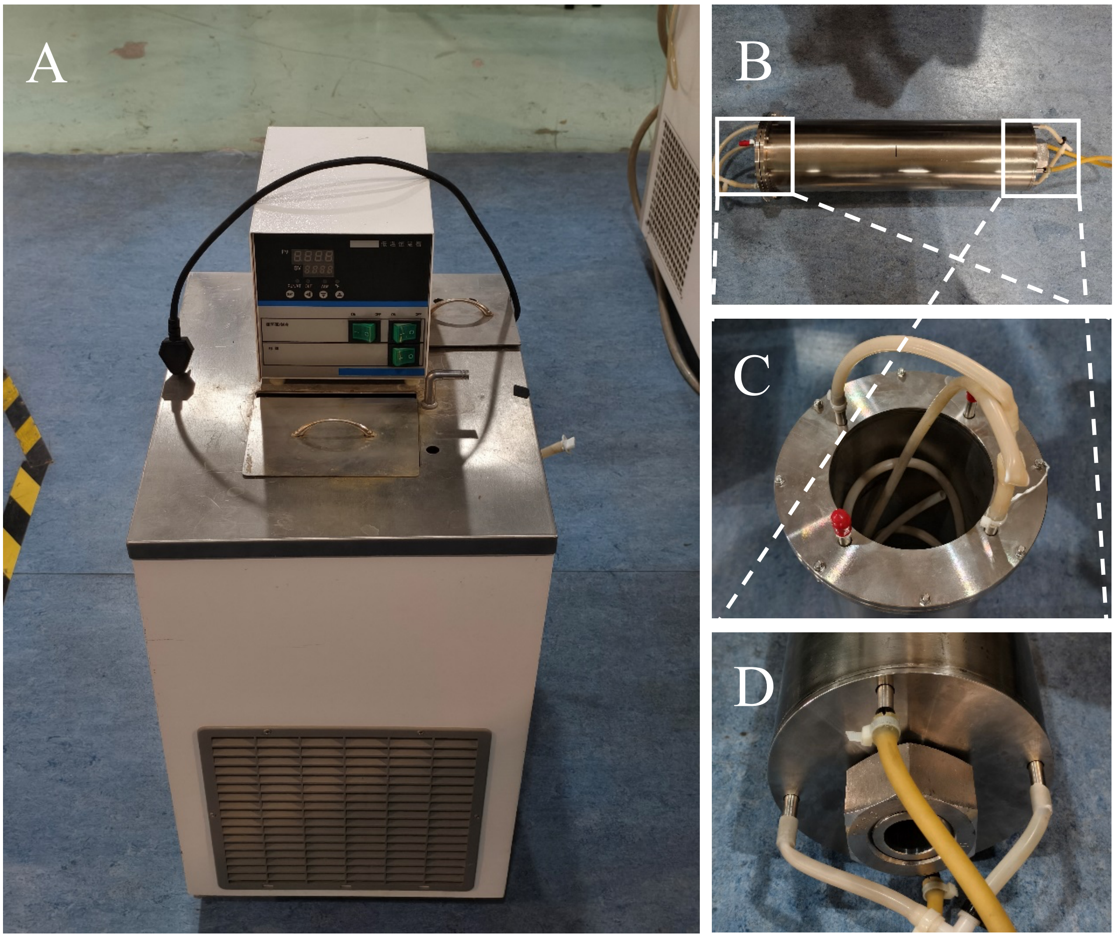

Figure S2. Photographs of the temperature control equipment, including the water bath temperature control unit (A) and the sample placement unit (B-D).

Figure S3

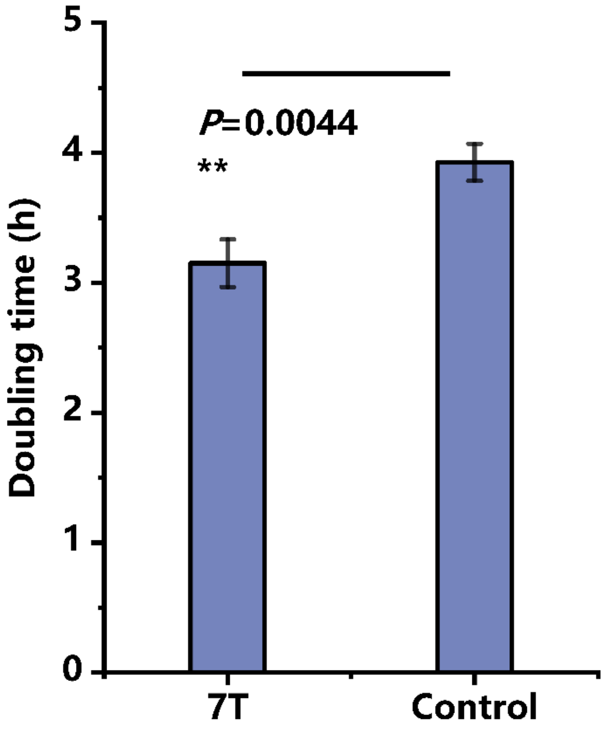

Figure S3. The doubling time of MSR-1 under a 7 T magnetic field and geomagnetic field (control) conditions, calculated from the 12-14 hour time interval. The number of samples in each group was three (n=3). The *P* value was calculated using the two-sided unpaired Student's *t*-tests. $^{**}P < 0.01$.

Figure S4

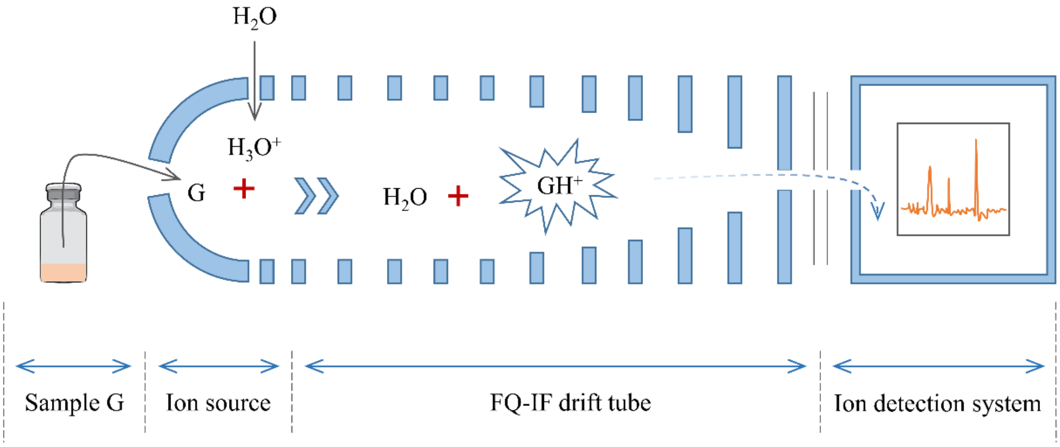

Figure S4. Schematic diagram of the PTR-MS system, which is composed of an ion source, an FQ-IF drift tube, and an ion detection unit. Sample G represents the target gas introduced for detection.

Table S1. Primers used in this study

Primer	Sequence (5′-3′)	Description
napF-F	GATGTCGCACAGCCTTAGC	RT-PCR for *napF* gene
napF-R	CGGCATCGATCAAAGGGATG	RT-PCR for *napF* gene
nirT-F	CCATTCACTACACCAACCGTTC	RT-PCR for *nirT* gene
nirT-R	ATGGCAGTTGCGGCATTC	RT-PCR for *nirT* gene
norC-F	CGGTGTTCGTTGCCTTGA	RT-PCR for *norC* gene
norC-R	CAGACATTGCCCAGTTCCG	RT-PCR for *norC* gene
nosZ-F	TCGCCACGGTGTCCTTT	RT-PCR for *nosZ* gene
nosZ-R	ATCACCTGACCGCTTTGGC	RT-PCR for *nosZ* gene
rpoC-F	ATCTGGTCTACCGCCATTG	RT-PCR for *rpoC* gene
rpoC-R	CCTTGCCGAACGAAATACC	RT-PCR for *rpoC* gene

Table S2. Upregulated and downregulated genes of transcriptome

Upregulated Gene ID	Downregulated Gene ID
MGMSRv2__1819	MGMSRv2__2794
MGMSRv2__1818	MGMSRv2__0533
MGMSRv2__1998	MGMSRv2__2796
MGMSRv2__1434	MGMSRv2__1712
MGMSRv2__3972	MGMSRv2__1352
MGMSRv2__2504	MGMSRv2__2797
MGMSRv2__0438	MGMSRv2__2695
MGMSRv2__2570	MGMSRv2__4247
MGMSRv2__0875	MGMSRv2__3298
MGMSRv2__3971	MGMSRv2__3299
MGMSRv2__0726	MGMSRv2__0364
MGMSRv2__3969	MGMSRv2__1536
MGMSRv2__1426	MGMSRv2__0477
MGMSRv2__1714	MGMSRv2__0471
MGMSRv2__2192	MGMSRv2__0146
MGMSRv2__3974	MGMSRv2__0815
MGMSRv2__2914	MGMSRv2__2017
MGMSRv2__0879	MGMSRv2__1571
MGMSRv2__3747	MGMSRv2__0449
MGMSRv2__2748	MGMSRv2__0115
MGMSRv2__0380	MGMSRv2__1260
MGMSRv2__3196	MGMSRv2__0468
MGMSRv2__1397	MGMSRv2__1259
MGMSRv2__2644	MGMSRv2__0470
MGMSRv2__1470	MGMSRv2__0581
MGMSRv2__2514	MGMSRv2__1033
MGMSRv2__3915	MGMSRv2__3899
MGMSRv2__2002	MGMSRv2__0365
MGMSRv2__2915	MGMSRv2__1510
MGMSRv2__0585	MGMSRv2__0185
MGMSRv2__2093	MGMSRv2__0357
MGMSRv2__0595	MGMSRv2__0848
MGMSRv2__2006	MGMSRv2__2887
MGMSRv2__1703	MGMSRv2__2706
MGMSRv2__0557	MGMSRv2__2694
MGMSRv2__2003	MGMSRv2__3836
MGMSRv2__2571	MGMSRv2__0469
MGMSRv2__0250	MGMSRv2__1713
sRNA0220	MGMSRv2__1408
MGMSRv2__0855	MGMSRv2__1353

MGMSRv2__1399	MGMSRv2__2795
MGMSRv2__1404	MGMSRv2__1261
MGMSRv2__4192	MGMSRv2__1883
MGMSRv2__3992	MGMSRv2__0543
MGMSRv2__2843	MGMSRv2__0318
MGMSRv2__0734	MGMSRv2__1612
MGMSRv2__0586	MGMSRv2__1990
MGMSRv2__2646	MGMSRv2__2014
MGMSRv2__0883	MGMSRv2__0186
MGMSRv2__0273	MGMSRv2__1262
MGMSRv2__1799	MGMSRv2__0467
MGMSRv2__1425	MGMSRv2__0306
MGMSRv2__0544	MGMSRv2__1389
MGMSRv2__0980	MGMSRv2__0404
MGMSRv2__3174	MGMSRv2__2772
MGMSRv2__2656	MGMSRv2__0346
sRNA0043	MGMSRv2__2221
MGMSRv2__1698	MGMSRv2__0976
MGMSRv2__2490	MGMSRv2__2311
MGMSRv2__1394	MGMSRv2__0486
MGMSRv2__1307	MGMSRv2__3619
MGMSRv2__0959	MGMSRv2__0145
sRNA0226	MGMSRv2__0065
MGMSRv2__1396	MGMSRv2__3506
sRNA0209	MGMSRv2__3835
MGMSRv2__1395	MGMSRv2__3702
MGMSRv2__1959	MGMSRv2__2926
MGMSRv2__2623	MGMSRv2__1409
MGMSRv2__0234	MGMSRv2__2769
MGMSRv2__1393	MGMSRv2__4038
MGMSRv2__4016	MGMSRv2__0439
MGMSRv2__3537	MGMSRv2__3979
MGMSRv2__0457	MGMSRv2__1263
sRNA0136	MGMSRv2__2792
MGMSRv2__0876	MGMSRv2__1720
MGMSRv2__4204	MGMSRv2__3759
MGMSRv2__0725	MGMSRv2__3861
MGMSRv2__2682	MGMSRv2__1603
MGMSRv2__0333	MGMSRv2__0114
MGMSRv2__2649	MGMSRv2__1694
MGMSRv2__1431	MGMSRv2__1995
MGMSRv2__2942	MGMSRv2__0838
MGMSRv2__0863	MGMSRv2__2194
MGMSRv2__1398	MGMSRv2__1258

MGMSRv2__4229	MGMSRv2__2774
MGMSRv2__0160	MGMSRv2__3200
MGMSRv2__1369	MGMSRv2__3703
MGMSRv2__3970	MGMSRv2__3813
MGMSRv2__1913	MGMSRv2__0347
MGMSRv2__2624	MGMSRv2__0914
MGMSRv2__2833	MGMSRv2__2484
MGMSRv2__0409	MGMSRv2__0368
MGMSRv2__4022	MGMSRv2__0972
MGMSRv2__3726	MGMSRv2__2773
MGMSRv2__3724	MGMSRv2__3508
MGMSRv2__0977	MGMSRv2__2779
MGMSRv2__1999	MGMSRv2__2482
MGMSRv2__0266	MGMSRv2__3984
MGMSRv2__2666	MGMSRv2__2793
MGMSRv2__3175	MGMSRv2__3034
MGMSRv2__0684	MGMSRv2__3982
MGMSRv2__2400	MGMSRv2__1611
MGMSRv2__4203	MGMSRv2__4079
MGMSRv2__2444	MGMSRv2__2108
MGMSRv2__2755	MGMSRv2__0428
MGMSRv2__1896	MGMSRv2__2924
MGMSRv2__3262	MGMSRv2__2782
MGMSRv2__2784	MGMSRv2__0550
MGMSRv2__0006	MGMSRv2__4127
MGMSRv2__3723	MGMSRv2__0512
sRNA0135	MGMSRv2__4244
MGMSRv2__2809	MGMSRv2__2775
MGMSRv2__0167	MGMSRv2__2013
MGMSRv2__1787	MGMSRv2__2771
MGMSRv2__3905	MGMSRv2__1034
MGMSRv2__4128	MGMSRv2__3159
MGMSRv2__1755	MGMSRv2__1871
MGMSRv2__2742	MGMSRv2__3701
MGMSRv2__1775	MGMSRv2__1215
MGMSRv2__3050	MGMSRv2__0363
MGMSRv2__3725	MGMSRv2__0935
MGMSRv2__3973	MGMSRv2__0456
MGMSRv2__3752	MGMSRv2__3981
MGMSRv2__0794	sRNA0123
MGMSRv2__0578	sRNA0049
MGMSRv2__3753	MGMSRv2__2781
MGMSRv2__3623	MGMSRv2__0113
sRNA0005	MGMSRv2__2770

MGMSRv2__3811	MGMSRv2__3113
MGMSRv2__3779	MGMSRv2__0316
MGMSRv2__1938	MGMSRv2__2327
MGMSRv2__0078	MGMSRv2__0995
MGMSRv2__0569	MGMSRv2__2352
MGMSRv2__0565	MGMSRv2__0500
MGMSRv2__1093	MGMSRv2__4071
MGMSRv2__3036	MGMSRv2__2778
MGMSRv2__2328	MGMSRv2__2154
MGMSRv2__3536	MGMSRv2__2889
MGMSRv2__3728	MGMSRv2__2885
MGMSRv2__0626	MGMSRv2__1354
MGMSRv2__1253	MGMSRv2__2472
MGMSRv2__3533	MGMSRv2__1476
MGMSRv2__3058	MGMSRv2__3108
MGMSRv2__0658	MGMSRv2__3562
sRNA0128	MGMSRv2__0302
MGMSRv2__0932	MGMSRv2__4233
MGMSRv2__1946	MGMSRv2__0184
MGMSRv2__1293	MGMSRv2__0307
MGMSRv2__1918	MGMSRv2__3039
MGMSRv2__3791	MGMSRv2__0426
sRNA0223	MGMSRv2__3980
MGMSRv2__0093	sRNA0166
MGMSRv2__0651	MGMSRv2__1135
MGMSRv2__2076	MGMSRv2__0759
MGMSRv2__1815	MGMSRv2__2016
MGMSRv2__1943	MGMSRv2__2354
MGMSRv2__3762	MGMSRv2__2130
MGMSRv2__1980	MGMSRv2__0112
MGMSRv2__0351	MGMSRv2__2903
MGMSRv2__0106	MGMSRv2__0427
MGMSRv2__1705	MGMSRv2__2639
MGMSRv2__2548	MGMSRv2__3983
MGMSRv2__3087	MGMSRv2__2060
sRNA0054	MGMSRv2__3793
sRNA0134	MGMSRv2__2994
MGMSRv2__2535	MGMSRv2__0767
	MGMSRv2__1770
	MGMSRv2__0422
	MGMSRv2__3700
	MGMSRv2__0548
	MGMSRv2__0107
	MGMSRv2__0362

	MGMSRv2__0325
	MGMSRv2__2111
	MGMSRv2__0361
	MGMSRv2__3962
	MGMSRv2__3715
	MGMSRv2__3963
	MGMSRv2__2015
	MGMSRv2__0424
	MGMSRv2__0348
	MGMSRv2__2153
	MGMSRv2__4019
	MGMSRv2__2278
	MGMSRv2__2018
	MGMSRv2__0747
	sRNA0118
	sRNA0100
	MGMSRv2__2061
	MGMSRv2__2835
	MGMSRv2__3333
	MGMSRv2__3114
	MGMSRv2__1654
	sRNA0003
	MGMSRv2__1901
	sRNA0184
	MGMSRv2__3859
	sRNA0089
	MGMSRv2__2242
	MGMSRv2__3697
	sRNA0076
	sRNA0235
	sRNA0221
	MGMSRv2__3067
	MGMSRv2__0105
	MGMSRv2__1614
	MGMSRv2__1406
	MGMSRv2__1915
	sRNA0153
	MGMSRv2__0328
	MGMSRv2__1912
	MGMSRv2__0680
	MGMSRv2__2201
